# Attitude, Practice and Barriers of Academic Research among Radiology Residents in Ethiopia: A Cross-Sectional Survey

**DOI:** 10.4314/ejhs.v32i1.10S

**Published:** 2022-10

**Authors:** Abdi Dandena, Tesfaye Kebede

**Affiliations:** 1 Department of Radiology, Faculty of Medical Science, Institute of Health, Jimma University, Ethiopia; 2 Department of Radiology, Faculty of Medical Science, Institute of Health, Addis Ababa University, Ethiopia

**Keywords:** Attitude, perception, barriers, Research, Radiology Residents

## Abstract

**Background:**

Radiology has become one of the most sought out residency programs in the country attracting highly qualified candidates from all over the country. The objective of the study was to determine the attitude, practice and barriers of radiology residents towards academic research:

**Method:**

A descriptive cross-sectional survey was conducted across all five institutions currently giving radiology residency programs in the country the survey was carried out from 12/16/2020 to 12/12/2021. Using a questionaries' that were distributed to the residents using google docs.

**Results:**

There were A total of 120 radiology residents participated in the study. 93(77.5%) of the participants were male while the rest 27 (22.5%) were female. With the mean age of the participants was 29 (1.75)years of age. Out of the 120 residents only 6 (5%) of them have published a research paper. 92.6% of the respondents were found to have a positive attitude towards academic research. Some of the major barrier to research identified by the study were the lack of time due to heavy workload, lack of Training course, inadequate Mentor Support and inadequate financial support.

**Conclusions:**

The study has showed that the overall attitude of radiology residents across the country towards academic research was positive but the practice of the residents in various academic research activities was found to be low. The authors recommend that all the stakeholders in radiology post graduate education to encourage residents in their academic pursuits by providing the necessary time and resources needed to perform quality research

## Introduction

Medical research is a systematic process implemented to get new knowledge on disease causation, diagnosis and treatment and it's an important component in the development of medical science and health care and is critical for keeping up with the rapidly evolving field of medicine. For these reasons many believe that research should be integrated in the curriculum of medical education(1). However, health research has been a low priority area in the developing world, mostly due to lack of the necessary infrastructure needed to cultivate research minded competent health professionals. Additionally working in Medical research has not been not being a fiscally rewarding venture for most health professionals working in developing countries (2).

**Figure 1:**
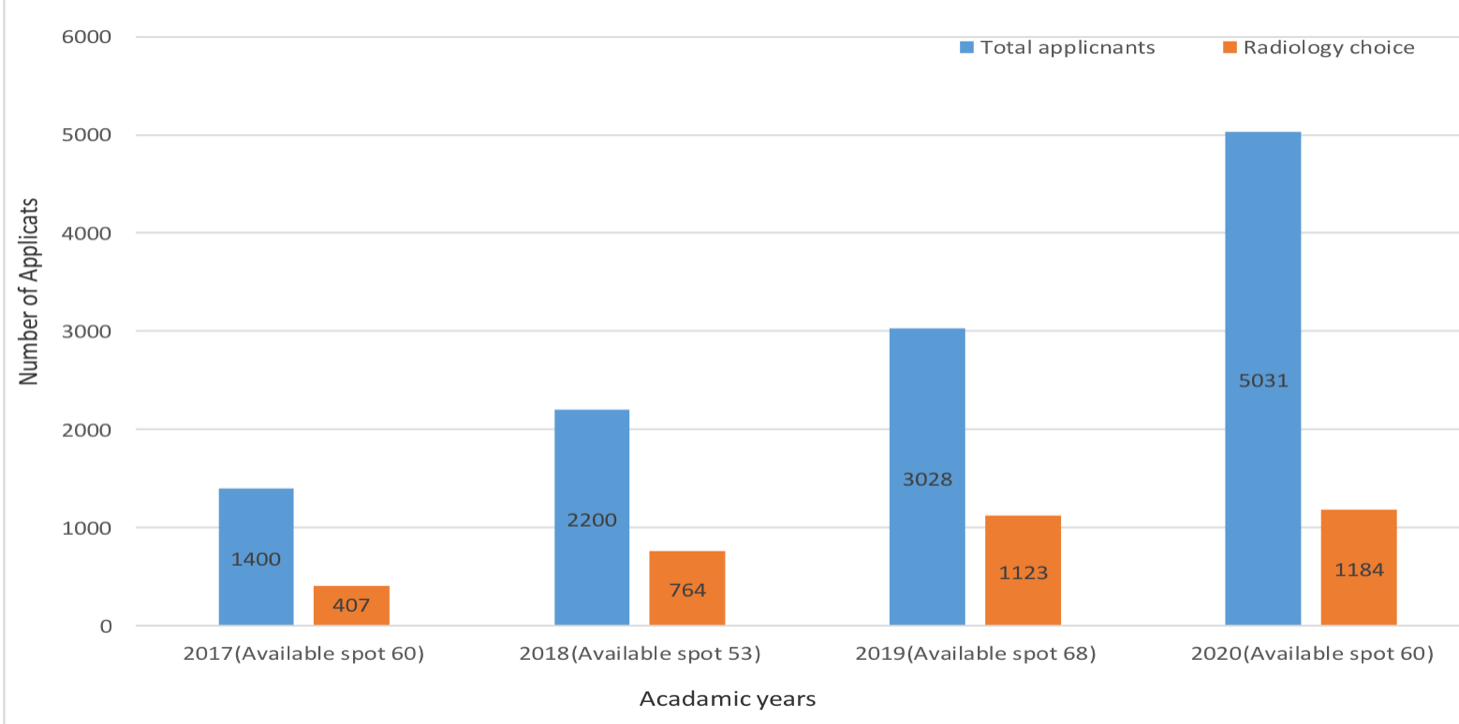
The number of total applicants versus Radiology residency applicants over the past four Ethiopian Residency matching Exams (ERMP) from 2017–2020.

Radiology is currently one of the most competitive residency programs in Ethiopia attracting highly talented and accomplished candidates form across the country. Since the initiation of the Ethiopian residency matching program (ERMP) back in 2017, out of the 20 different residency programs offered across the country radiology has constantly been the number one desired residency program in the country with the number of applicants exceeding the number of available spots by many folds (Figure 1). Consequently, the pool of radiology residents currently enrolled in the different programs across the country are more talented and academically minded than ever before. With many of the higher scorers in medical school joining the field of radiology.

Usually when highly motivated residents are engaged in clinical practice, they will be quick to recognize that there are gaps that are apparent in their day-to-day practice be it a knowledge gap or lack of facilities which prevents the delivery of the much needed care to the patients. This is more so in residents practicing in areas with limited setups like low income countries(3). Although this observation holds a great promise for the field of radiology in the country, having highly qualified residents in the field of radiology does not necessarily equate to having competent Clinical researchers.

## Methods

**Study design, study area and study period**: A descriptive cross-sectional survey was conducted across all five institutions currently providing radiology residency programs in the country which included Addis Ababa University, University Gondor, Mekelle University, St Paul Hospital Millennium Medical College, and Bahirdar University. The survey was carried out from 12/16/2020 to 13/6/2021.

**Study population and sampling method**: All willing residents which were enrolled at one of the Radiology Residency programs across the country during the study period were considered as the study population. During the study period there were a total of 175 Radiology Residents studying across the country hence a confidence interval of 95% a sample size of 120 was reached.

**Data collection**: A pretested structured questionnaire’ was used to collect the data. The questionnaire was adopted from a validated questionnaire by vodopivec,(1) and was modified to be relevant to radiology residents. The questionnaire had been adopted by similar studies in the country as well as other developing nations.(1) The questionnaire was pre tested on 5 radiology residents studying at Addis Ababa University and had undergone slight modification following feedbacks received from the residents. The questions in the questionaries' were then converted to digital form using google Docs website and the websites links was sent to all the residents across the country using shared social media platforms including personal emails and telegram accounts for better access

The questionnaire consisted of three parts: residents' profiles which include sociodemographic data, educational background and research backgrounds. The second part consist of attitude regarding research and consist of 9 questions that are answerable using the 5-point Likert scale. The questions were randomized to combat any bias that will be introduced by order and reverse coded questions were placed to detect any inconsistent response during the survey. And respondent's which had inconsistencies in their response were excluded during the Data analysis (4,5).

The third part consist of research practice and barriers experienced by residents. This segment consisted of ‘yes’ and ‘no’ questions and multiple choice questions which assessed the resident's prior exposure in various academic research activities. In Additon, it tried to asses the difficulties faced by residents in their academic pursuits.

**Operational definitions**

**Attitude:** The feeling of the participants towards the scientific inquiry process, statistics, literature review and critical appraisal of evidence(5).

**Practice**: previous engagement in any research endeavors such as prior publications, presentations, case reports and participation in activities like workshops for research methodology(6).

**Attitude score**: from the nine Likert score questions which assessed attitude a mean score was calculated and a score of 0.01–1.0 was regarded as having a strong disagreement, from 1.01 to 2.0 was regarded as disagreement and from 2.01–3.0 was regarded as neutral, from 3.01–4.0 was regarded as agreement and from 4.01–5.0 as strong agreement. And residents with a mean attitude score above 3.0 were considered as having a positive Attitude towards academic research during residency and residents which had a score of less than 3.0 were considered to have a negative attitude towards academic research during residency.

**Data analysis**: Data was downloaded from the google doc's website into an Excel spreadsheet and was converted into a Spss dataset where it was proof read manually to maintain the quality of the data. The data was then analyzed using an IBM Spss v24 software. The data was presented using graphs and tables. Socio-demographic data were analyzed by frequency, proportions, mean, median and standard deviation Likert scale items were calculated using a mean score from the five Likert-type items.

**Ethical consideration**: The study was approved by the Radiology Department Research and Ethics Committee. Participation was fully based on volunteerism, and written consent was included with the questionnaire. The names and other unique identifiers of the participants were not used for the sake of confidentiality.

## Results

There were a total of 175 radiology residents during data collection and a total of 120 radiology residents participated in the study. 93(77.5%) of the participants were male while the rest 27 (22.5%) were female. With the mean age of the participants was 30 years of age, 35(29.2%) of the participants were married. The majority of the study participants were final year residents. sixty eight (56.3%) of the residents were sponsored from Academic institutions while the other 52(43.3%) residents were sponsored from regional hospitals (Table 1).

**Table 1:** background information of radiology residents who participated in the study

Age	Number	percent
< 25 years	3	2.5
25–29 years	62	51.7
30–34 years	53	33.3
>35 years	2	1.7
Sex		
Male	93	77.5
Female	27	22.5
Marital Status		
Married	35	29.2
Single	85	70.8
Year of Residency		
R1	21	17.5
R2	33	27.5
R3	66	55.0
Sponsoring institutions		
Academic institutions	68	56.7
Regional Hospitals	52	43.3
Mode of Undergraduate		
Learning		
Problem based learning	16	13.3
Lecture based learning	103	85.8
Previous exposure to research in undergraduate studies		
Performed a group research	85	70.8
Performed a solo research	35	29.2

Out of the 120 residents only 6 (5%) of them have published a research paper and from the residents with previous publication only three had more than one publication. 14(11.7%) of the residents have participated in research activities in addition to mandatory dissertation. 12(10%) of the residents have participated in the writing of a case report during their residency program. Only one resident has presented his research paper on a conference, which was at a regional level. 54(45%) of the residents read academic journals at a regular basis. 18(15%) of Residents have participated in a research methodology workshop in the past (Table 2).

**Table 2:** Research practice among radiology residents who participated in the study

Questions	Yes N (%)	No N (%)
Have you published a research paper before?	6(5)	114(95)
Have you presented your research in a conference?	1(0.8)	119(99.2)
Have you worked on a case report during your residency?	12(10)	108(90)
Have you worked on a research project other than the mandatory dissertation during your residency?	14(11.7)	106(88.3)
Do you read scientific journals regularly?	54(45)	66(55)
Have you participated in a critical appraisal of research paper?	12(10)	108(90)
Have you ever participated in a research methodology workshop?	18(15)	101(85)
Have you ever written a grant proposal for a research project?	55(45.8)	65(54.2)

On the questions regarding attitude of radiology residents regarding academic research from the 120 residents 16 (13.3%) of the respondents were omitted due to inconsistent answers. From the 104 valid responses most of the residents 102(96.2%) were shown to have a positive attitude towards academic research with a mean attitude score of 3.78 (Table 3).

**Table 3:** Attitude of residents regarding academic research practice in radiology residency (N = 104)

Questions	Strongly Disagree N (%)	Disagree N (%)	Neutral N (%)	Agree N (%)	Strongly agree N (%)		
Training in medical research methodology should be included in radiology residency program	2(1.9)	0	6(5.7)	40(37.7)	58(54.7)	Attitude score	
Engaging in medical research can improve patients outcome	1(1)	0	4(3/8)	36(34.6)	63(60.6)	< 3.00 N (%)	>3.00 N (%)
Engaging in research increase the burden on radiology Residents	3(2.8)	19(17.9)	19(17.9)	41(38.7)	24(22.6)	4 (3.8)	102 (96.2)
Research should be mandatory for appearing in final exams in post graduate studies	10(9.4)	29(27.4)	22(20.8)	33(31.1)	12(11.3)	Mean attitude score 3.78	
Research time should be allotted separately during radiology residency training	0	2(1.9)	8(7.5)	46(43.4)	50(47.2)		
Radiology resident's should not engage in Research Activities	38(35.8)	52(49.1)	12(11.3)	2(1/9)	2(1.9)		
Radiology residents are given adequate training on Research methodology	26(24.5)	30(28.3)	44(41.5)	5(4.7)	1(0.9)		
I am confident in my abilities to carrying out research in the field of radiology	4(3.8)	15(14.3)	30(28.6)	42(40)	14(13.2)		
I want to carry out Medical research In the field of radiology in the Future.	0	3(2.8)	15(1.2)	50(7.2)	38(35.8)		

**Fig 2:**
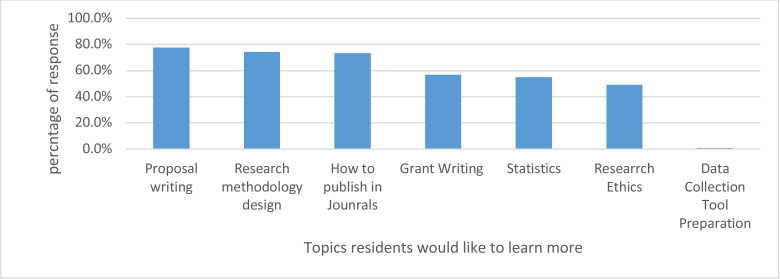
Aspect of research training that radiology residents would like to learn more.

Regarding the topic of research training, The number one area residents would like to improve on was on proposal writing followed by research methodology design, how to publish in journals and how to write research grants. (Figure 2).

**Fig 3:**
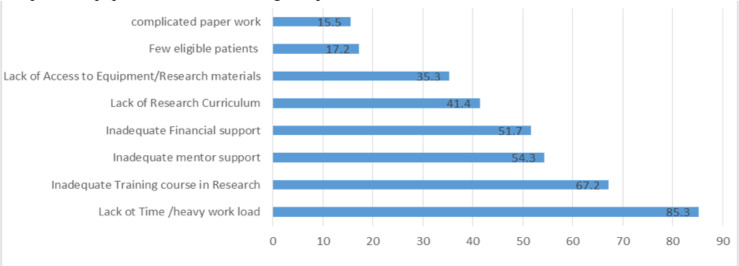
Barriers and Difficulties faced by Radiology residents in conducting Research.

Some of the major barriers and difficulties that were faced by the resident that were identified by the study were the lack of time due to heavy workload which was an issue for 99(85.3%) of the residents, followed by lack of Training course 78(67.2%), inadequate Mentor Support 63(54.3%), inadequate financial support 60(51.7%) as well as lack of access to equipment's or research materials, complicated paper work , lack of eligible patients for their research and lack of acknowledgment for their work (Figure 3).

There was a statistically significant association found between being educated at Jimma Universityin undergraduate studies and publishing a research paper in a journal. Practice of publishing was not found to have an association with gender, marital status, mode of learning and type of sponsoring institution (Table 4).

**Table 4:** Factors affecting publication outcomes in radiology residents in Ethiopia

Factors assessed	Publication outcomes			
	
	Number of Residents that published	Number of Residents that didn't publish	P- Value	Phi (Φ)
Gender				
Male	5	88	0.812	
Female	1	26		
Marital status				
Single	3	82	0.249	
Married	3	32		
Mode of learning				
Lecture based learning	6	97	0.594	
Problem based learning	0	16		
Sponsoring institution				
Academic institution	64	4	0.612	
Regional hospitals	50	2		
Undergraduate studies				
Jimma University	4	23	0.008	Φ 0.243
Other university	2	91		

## Discussion

The study showed that the overall attitude of radiology residents across the country towards academic research was positive, but the practice of the residents in various academic research activities was found to be low. These findings were similar to other studies conducted in the country. In a study conducted at Black lion hospital, Addis Ababa university 344 residents from all the different departments in the hospital were evaluated and the study showed an overall positive attitude towards an academic research(1). Some of the possible reasons that could explain the disparity between the overwhelmingly positive attitude towards academic research and but the low level of practice could be due to the medical educational curriculum in Ethiopia, which is very heavily oriented towards theoretical teaching and research is not given due attention in both undergraduate and post graduate studies(7).

The study did not find any gender and other socio-demographic finding which affected the residents' Attitude and practice towards academic research but has found that there were more than three times male residents compared to female residents. This disparity can possibly be explained by the fewer female medical doctors in the country secondary to various cultural and social factors.(10)

The study has shown that only few residents have published a research paper in the past and fewer were able to present their paper In conference and this finding is seen in many studies globally where disparity has been show between positive attitude towards research and actual participation in research among health care professionals.(11) This disparities can be addressed by integrating research initiatives and competitions into the residency programs to promote the residents engage in research activities. All stakeholders including the universities and the professional organizations including the Radiology society of Ethiopia should provide platforms for residents to present their research papers. It's been shown that residency programs that adopt active research programs significantly enrich the residents education.(3)

Our study has found the only factor shown to be statistically significantpertaining to resident's publication outcomes was having an undergraduate training at Jimma University. This May partly be due to the emphasis given at Jimma university towards research evidenced by engagement of undergraduate students in community based training programs (CBTP) during their stay at medical school and a Team training program (TTP) in their internship year which is are programs which are heavily research oriented. These programs allows students to participate in the collection, analysis and interpretation of data throughout their medical training, which is not practiced in most medical institutions inthe author's experience.(12) One of the main difficulties faced by residents in conducting research was the lack of time due to a heavy workload from their day to day work and many studies have shown that giving residents a protected time to critically read and understand the published literature and attend methodology lectures can positively correlate with a resident's research productivity.(13,14,15)

Other important barriers identified in the study were inadequate mentor support, lack of training, inadequate financial support and lack of recognition for their hard work. These factors were also noted in a study conducted with dermatology residents and has been shown to cause residents to lose interests in perusing academic carriers.(16) Similarly, limited time, poor research training, inadequate source of funding and inadequate mentor support were also found to be major hurdles for academic research in studies conducted in India, Pakistan and Saudi Arabia. (17–19)

Although it may not be possible to fully address all the barriers that affect resident's research productivity studies have found simple research enhancing strategies including a regular journal club, allotting a dedicated protected time for research, hiring of research faculty, initiating research awards, recognitions, grant competitions and organizing platforms were residents can present their findings in annual conferences and meetings has been shown to increases the research production of residents in radiology departments. The study also highlighted that most residents have not presented their researches in academic conferences. These may be secondary to lack of platforms for residents to present research in their institutions as well as nationally.

There is a positive attitude among current radiology residents for academic research but the practice and engagement in any academic research activities is very low. Several barriers faced by radiology residents were identified in the study including lack of time, lack of training, inadequate financial and mentor support. The authors recommend that all Training institutions should revise the time allocated for research, if there is any, and try to provide sufficient time. Additionally institutions should also assign and monitor mentors for optimal support of the residents' research work which in author's opinion improves the research output. There should be programs in place to incentivize high impact research among Residents. Additionally platforms should be provided for residents to present their finding to their colleagues
